# Retraction Note: 8-bromo-7-methoxychrysin suppress stemness of SMMC-7721 cells induced by co-culture of liver cancer stem-like cells with hepatic stellate cells

**DOI:** 10.1186/s12885-023-11698-1

**Published:** 2023-12-07

**Authors:** Qi Wen, Chang Xu, Jie Zhou, Nuo-Min Liu, Ying-Hong Cui, Mei-Fang Quan, Jian-Guo Cao, Kai-qun Ren

**Affiliations:** 1 Department of Pharmaceutical Science, Medical College, Hunan Normal University, Changsha, 410013 China; 2 Key Laboratory of Study and Discover of Small Targeted Molecules of Hunan Province, Changsha, 410013 China

**Retraction Note**: ***BMC Cancer* 19, 224 (2019)**

10.1186/s12885-019-5419-5

The Editors have retracted this article because of significant concerns with a number of figures, specifically:

- Figure 7d, CD133 panel, appears to be the same as Fig. 2D, Oct 4 panel, in [1]
- Figure 4b, FAP-a panel, appears to be the same as Fig. 3D, p53 panel, in [2]
- Figure 3a, b-actin panel, appears to be the same as Fig. 3K, b-actin panel, in [3]

The authors were contacted and asked for an explanation but did not provide one. The Editors therefore no longer have confidence in the integrity of the data in this article.

Kai-qun Ren agrees with the retraction. None of the other authors have responded to any correspondence from the Editors about this retraction.
